# ﻿One new species of *Stegocephalus* Krøyer, 1842 (Amphipoda, Stegocephalidae) described from a seamount of the Caroline Plate, NW Pacific

**DOI:** 10.3897/zookeys.1195.114209

**Published:** 2024-03-14

**Authors:** Yanrong Wang, Zhongli Sha, Xianqiu Ren

**Affiliations:** 1 Department of Marine Organism Taxonomy and Phylogeny, Institute of Oceanology, Chinese Academy of Sciences, Qingdao 266071, China; 2 College of Biological Sciences, University of Chinese Academy of Sciences, Beijing 100049, China; 3 Laoshan Laboratory, Qingdao 266237, China; 4 Shandong Province Key Laboratory of Experimental Marine Biology, Institute of Oceanology, Chinese Academy of Sciences, Qingdao, 266071, China

**Keywords:** Deep sea, morphology, new species, Stegocephalinae, taxonomy

## Abstract

A new species of the subfamily Stegocephalinae, *Stegocephaluscarolus***sp. nov.**, is described from a seamount in the Caroline Plate. Two related species, *S.cascadiensis* (Moore, 1992) and *S.longicornis* (Gurjanova, 1962), were previously reported in the North Pacific. Important morphological characters which differentiate *S.carolus***sp. nov.** from *S.cascadiensis* are found in antenna 1, the mouthparts, pereopod 7 and the length of rami of uropods 2 and 3. The new species differs from *S.longicornis* by characters of antenna 1, the mouthparts and the shape of epimeral plate 3. Additionally, the morphological differences between the new species and the remaining seven species of *Stegocephalus* are also presented.

## ﻿Introduction

The family Stegocephalidae Dana, 1852 was revised by Berge and Vader (2001), and divided into five subfamilies: Andaniexinae Berge & Vader, 2001, Andaniopsinae Berge & Vader, 2001, Bathystegocephalinae Berge & Vader, 2001, Parandaniinae Berge & Vader, 2001 and Stegocephalinae Dana, 1852. Of these, Stegocephalinae is the largest, comprising 14 genera with 49 species (Horton et al. 2023), characterized by the presence of gaping and geniculate outer plate of maxilla 2, the flagellum of antenna 1 composed of not more than 10 articles and a telson that is longer than broad (Berge and Vader 2001). As the type genus of the Stegocephalinae, *Stegocephalus* Krøyer, 1842 can be distinguished from the other 13 genera by having the flagellum article 1 of antenna 1 not longer than the peduncle, the flagellum of antenna 2 with more than 10 articles and the palp of the maxilla 1 two-articulate (Barnard and Karaman 1991; Berge and Vader 2001). There are nine valid species of *Stegocephalus*, which are found in the Arctic, Southern Ocean (Antarctica), Atlantic, southern Mediterranean Sea and North Pacific (Berge and Vader 2001).

During a biodiversity survey of seamounts on the Caroline Plate in the northwest Pacific in 2019, conducted by the Chinese research vessel KEXUE, several individuals referable to *Stegocephalus* were collected. These specimens exhibit some distinctive characteristics differentiating them from the other nine described *Stegocephalus* species, and they are described as a new species herein.

## ﻿Materials and methods

**Figure 1.**
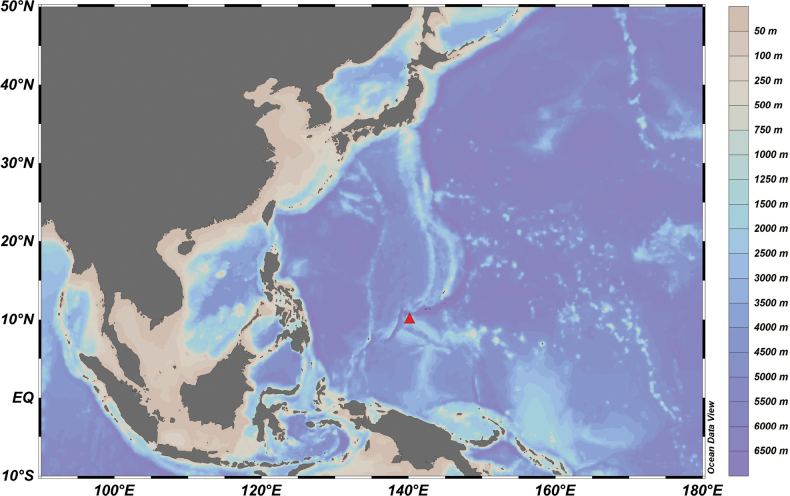
A map showing the location of the sampling site (red triangle) in the NW Pacific.

The present material was collected by ROV FAXIAN, during expeditions to seamounts (Fig. 1) on the Caroline Plate by the Institute of Oceanology, Chinese Academy of Sciences (IOCAS) during June and July 2019. The material was sorted on board and fixed in 96% ethanol, then transferred to 75% ethanol in the laboratory. The specimens are deposited in the Marine Biological Museum, Chinese Academy of Sciences, Qingdao, China. The specimens were examined and dissected with a dissecting microscope (ZEISS Discovery V20). Line drawings were completed using the software Adobe Photoshop CS6 with a graphics tablet. Length measurement was made along the outline of the animal, beginning from the rostrum to the posterior margin of the telson; total length of the specimens ranged from 3.4 to 8.9 mm. Additionally, COI (PP188368), 16S rDNA (PP187329) and 18S rDNA (PP187328) sequences of *Stegocephaluscarolus* sp. nov. were obtained from its mitochondrial genome by homologous alignment.

## ﻿Systematics

**Order Amphipoda Latreille, 1816**

**Suborder Amphilochidea Boeck, 1871**

**Superfamily Stegocephaloidea Dana, 1852**

**Family Stegocephalidae Dana, 1852**

**Subfamily Stegocephalinae Dana, 1852**

### ﻿Genus Stegocephalus

Taxon classificationAnimaliaAmphipodaStegocephalidae

Krøyer, 1842

36A72A1A-BC94-5EB8-8CA6-7A9BAB7F7EAE

#### Diagnosis.

Body smooth. Antenna 2 with peduncular article 4 shorter or longer than article 5. Epistomal plate absent. Mandible incisor lateral, toothed; left lacinia mobilis powerful, toothed, distally produced. Maxilla 1 palp well developed, 1- or 2-articulated. Outer plate of maxilla 2 gaping and geniculate, with setae distally with hooks (absent in *S.ampulla*). Palp of maxilliped 4-articulated, article 2 unproduced or inner margin produced distally. Pereopods 1–2 simple. Pereopod 6 basis conspicuously expanded. Uropod 3 biramous, outer ramus l-articulate, peduncle shorter than rami. Telson elongate, cleft (amended after Barnard and Karaman 1991; Berge and Vader 2001).

### ﻿ Stegocephalus carolus sp. nov.

Taxon classificationAnimaliaAmphipodaStegocephalidae

A02DE78F-014D-59BF-AAD3-175D6D33E819

https://zoobank.org/35CA4012-7AB1-4E86-BBC4-51A5B05F6EB2

Figs 2 , 3 , 4

#### Material examined.

***Holotype*.** MBM 286610, ♀ (8.9 mm, with 6 big yolks), dissected, unnamed seamount on Caroline Plate, NW Pacific, M5173, 10°03′N, 140°10′E, depth 1221 m, 30 May 2019. ***Paratypes***: MBM 287879, 2♂ (6.0 and 7.0 mm), not dissected, unnamed seamount on Caroline Plate, NW Pacific, M5117, 10°04′N, 140°12′E, depth 870–944 m, 28 May 2019; MBM 287878, 2♂ (7.6 and 3.4 mm), not dissected, unnamed seamount on Caroline Plate, NW Pacific, M5362, 10°04′N, 140°11′E, depth 813–1182 m, 2 June 2019.

#### Description.

First pereon segment longer than the rest; pleonite 1–3 dorsally smooth; epimeral plate 1 with posteroventral corner broadly rounded; epimeral plate 2 with posteroventral corner subacute; epimeral plate 3 without minute serration at posteroventral corner, posteroventral corner strongly produced, triangular. Head partially covered by pereonite 1 and coxa 1; much deeper than long. Rostrum absent. Eyes not apparent in ethanol-fixed material. Antenna 1 with callynophore well developed; peduncle about twice as long as first primary flagellar article; article 1 slightly longer than articles 1–2 combined; primary flagellum 7-articulate, article 1 longest; accessory flagellum 1-articulate, beyond article 1 of primary flagellum, bearing long setae apically. Antenna 2 slightly longer than antenna 1, peduncle (articles 3–5) slightly shorter than flagellum, article 4 longer than article 5, not setose; flagellum 12 articles, article 1 longer than rest of articles.

**Figure 2.**
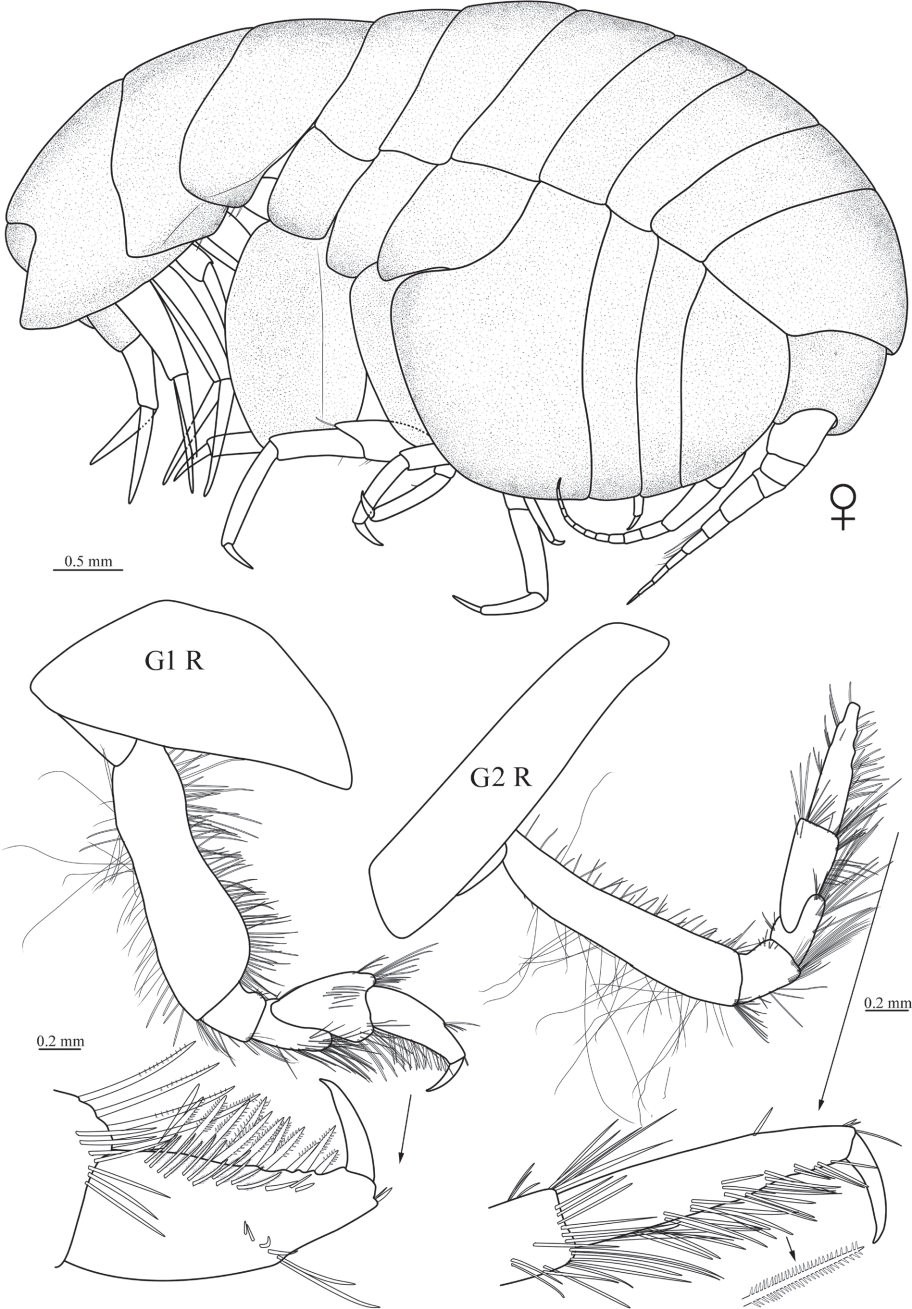
*Stegocephaluscarolus* sp. nov., MBM 286610, holotype, ♀ (8.9 mm): G1 R, right gnathopod 1; G2 R, right gnathopod 2.

***Mouthparts*.** Epistomal plate absent. Upper lip broader than long, bilobate, slightly asymmetrical. Lower lip with the narrow lobes bearing row of simple setae. Right Mandible incisor with 9 teeth; incisor of left mandible with 7 teeth, left lacinia mobilis with 9 teeth. Maxilla 1 palp 2-articulate, rectangular, apex not reaching above the apex of outer plate, apically bearing 4 plumose setae; outer plate with 9 setal-teeth arranged in a pseudocrown; inner plate with a well-developed shoulder, setae pappose. Maxilla 2 gaping and geniculate; outer plate with about 10 apical setae bearing distal hooks; inner plate with marginal and submarginal setae pappose. Maxilliped with inner plate subrectangular, not exceeding base of palp article 2, distal margin slightly concave, setose, medial setae-row present; outer plate only with inner margin bearing about 7 small robust setae; palp 4-articulate, article 2 distinctly shorter than article 1, inner margin produced distally, dactylus short, about 1/5 length of article 3.

Gills present on coxae 2–7. Oostegites present on 2–5.

**Figure 3.**
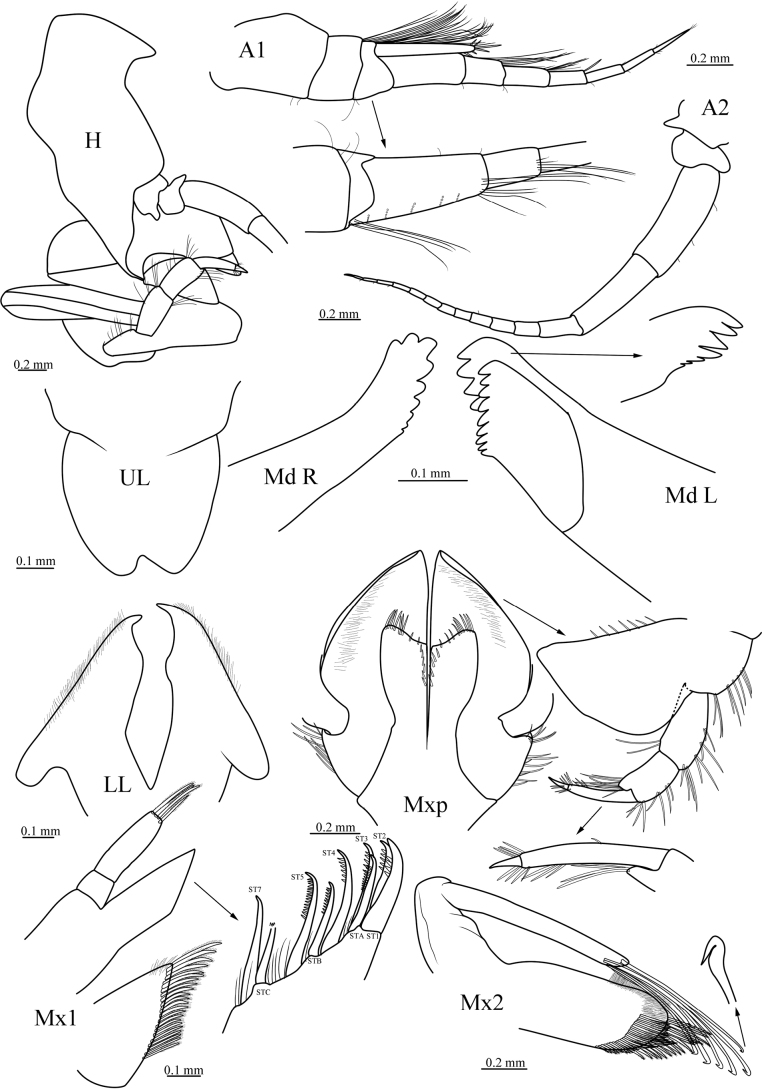
*Stegocephaluscarolus* sp. nov., MBM 286610, holotype, ♀ (8.9 mm): H, head; A1, antenna 1; A2, antenna 2; UL, upper lip; LL, lower lip; Md L, left mandible; Md R, right mandible; Mx1, maxilla 1; Mx2, maxilla 2; Mxp, maxilliped.

***Pereopods*.** Gnathopod 1 simple; coxa triangular, tapering, anterior margin convex; basis with anterior margin sinuous, both margins heavily setose; ischium not elongate, posterior margin bearing long setae, anterior margin with several short robust setae; carpus subequal in length to propodus, expanded distally, anterior margin bearing group of long setae distally, posterior margin setose; propodus tapering anterior margin bearing row of 6 robust setae about 2/3 length of propodus and two short robust setae subdistally, posterior surface rows of pectinate setae and rows of plumose setae; dactylus nearly straight, tapering, about 1/3 length of propodus. Gnathopod 2 simple; coxa much longer than broad, subrectangular, ventral margin slightly curved; basis much more slender than gnathopod 1, both margins setose; ischium slightly shorter than merus, subrectangular; carpus slender, not expanded distally, shorter than propodus, distal and posterior margin setose; propodus slender, setae on posterior surface similar to that of gnathopod 1; dactylus tapering, curved. Pereopod 3 coxa similar to that of gnathopod 2; basis linear, anterior margin bearing dense short robust setae, posterior margin bearing several long setae; ischium short; merus longer than carpus, anterodistally drawn out, with one long robust seta, posterior margin bearing several setae; carpus subequal in length to propodus, posterior margin with few setae; propodus with only posterior margin bearing few small setae; dactylus tapering, slightly curved. Pereopod 4 similar to pereopod 3, but coxa large, posteroventral lobe broadly rounded, posterior margin excavate. Pereopod 5 slightly longer than pereopod 4; coxa bilobate, posterior lobe deeper; basis linear, both margins setose; merus to dactylus of similar appearance to pereopod 4, but merus subequal in length to carpus, and propodus much slender, distinctly longer than carpus. Pereopod 6 longer than pereopod 5; coxa unilobate; basis expanded posteriorly, rounded below, anterior margin and anterior dorsal surface with several robust setae, posterior margin serrated with small setae; distal 5 articles of similar appearance to pereopod 5. Pereopod 7 shorter than pereopod 6, but longer than pereopod 5; basis expanded posteriorly, distinctly larger than that of pereopod 6, posterior margin serrated with small setae, beyond end of ischium; merus longer than carpus, margins setose; carpus shorter than propodus, anterior margin with three groups setae; propodus shorter than that of pereopod 6, anterior margin with small setae; dactylus slightly curved, tapering.

**Figure 4.**
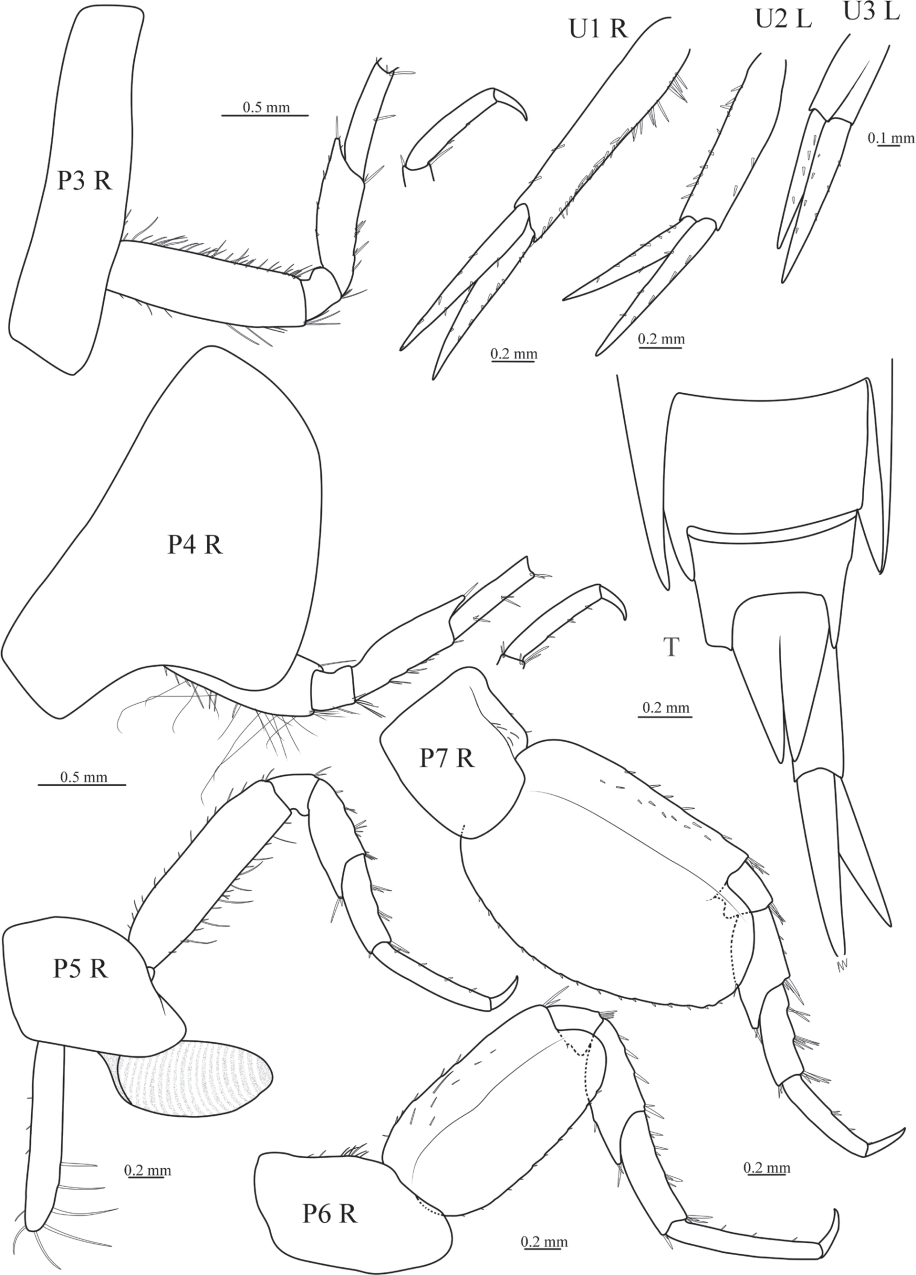
*Stegocephaluscarolus* sp. nov., MBM 286610, holotype, ♀ (8.9 mm): P3 R, right pereopod 3; P4 R, right pereopod 4; P5 R, right pereopod 5; P6 R, right pereopod 6; P7 R, right pereopod 7; U1 R, right uropod 1; U2 L, left uropod 2; U3 L, left uropod 3; T, telson.

***Uropods and telson*.** Uropod 1 peduncle 1.4 times longer than rami, outer margin densely setose, inner margin with only 2 robust setae; rami lanceolate, subequal in length, both rami with inner and outer margins setose. Uropod 2 shorter than uropod 1, peduncle subequal in length to rami, both margins setose; rami subequal in length, both rami with inner and outer margins setose. Uropod 3 shortest, peduncle distinctly shorter than rami, not setose; outer ramus 1-articulate, shorter than inner ramus, both rami with inner and outer margins setose. Telson longer than broad, without setae, cleft about 0.8 of total length, distally acute.

#### Etymology.

The new species is named after its type locality, the Caroline Plate.

#### Distribution.

Presently known only from a seamount of the Caroline Plate, at a depth of 813–1221 m.

#### Remarks.

According to the revision of the family Stegocephalidae Dana, 1852 by Berge and Vader (2001), *Stegocephaluscarolus* sp. nov. belongs to the subfamily Stegocephalinae Dana, 1852. The new species shares distinctive characteristics with other species in the genus *Stegocephalus* Krøyer, 1842, including a two-articulate palp of maxilla 1, a produced palp article 2 of the maxilliped, a conspicuously expanded basis of pereopod 6, a 1-articulate outer ramus of uropod 3, and a cleft telson. Two *Stegocephalus* species, *S.cascadiensis* (Moore, 1992) and *S.longicornis* (Gurjanova, 1962), have been previously reported in the North Pacific (Moore 1992; Berge and Vader 2001).

*Stegocephaluscascadiensis* (Moore, 1992) was originally reported from the Cascadia Abyssal Plain at a depth of 2740–2818 m (Moore 1992). *Stegocephaluscarolus* sp. nov. differs from *S.cascadiensis* by the peduncle of antenna 1 longer than the first flagellar article, and the accessory flagellum extending beyond the distal margin of the first flagellar article. Additionally, the apical setae on the outer plate of maxilla 2 are hooked in the new species, whereas, they are unhooked in *S.cascadiensis*. In *S.carolus* sp. nov., the first palp article of the maxilliped is subequal in length to the second article, whereas in *S.cascadiensis*, the first palp article is much longer than the second. The inner plate of the maxilliped is subrectangular with a concave distal margin in *S.carolus* sp. nov., whereas it is subtriangular in *S.cascadiensis*. The second palp article of the maxilliped in *S.carolus* sp. nov. has a produced inner margin, and the posterior margin of pereopod 7 is slightly serrate instead of smooth. Furthermore, the rami of uropod 2 are subequal in *S.carolus* sp. nov., whereas the inner ramus is longer than the outer ramus in *S.cascadiensis*. Lastly, the outer ramus of uropod 3 in *S.carolus* sp. nov. is shorter than the inner ramus, while in *S.cascadiensis*, it is longer than the inner ramus (Moore 1992, fig. 7).

*Stegocephaluslongicornis* (Gurjanova, 1962) was originally reported from the Bering Sea at a depth of over 2440 m. The new species differs from *S.longicornis* by: the shorter antenna 1, the 7-articulated primary flagellum and the 1-articulated accessory flagellum, while the primary flagellum is 11-articulate and the accessory flagellum is 3-articulate in *S.longicornis*; the distal margin of the inner plate of the maxilliped is concave rather than convex, and the inner margin of palp article 2 is produced, versus not produced in *S.longicornis*; and the posteroventral corner of epimeral plate 3 is strongly produced and triangular, while epimeral plate 3 is only produced in a small triangular angle in *S.longicornis* (Gurjanova 1962, fig. 130).

The remaining species of *Stegocephalus* are found in the Arctic, North Atlantic, or the Southern Ocean (Berge and Vader 2001). *Stegocephaluscarolus* sp. nov. can be easily distinguished from *S.abyssicola* (Oldevig, 1959) by its strongly projected posteroventral corner of the third epimeral plate (Oldevig 1959). It is also different from *S.ampulla* (Phipps, 1774), *S.inflatus* Krøyer, 1842, *S.rostrata* KH Barnard, 1932, and *S.similis* Sars, 1891 by the absence of a rostrum (Barnard 1932; Steele 1967; Berge and Vader 2001). Especially, it differs from *S.inflatus* by the pale of maxillae 1 being 2-articulated, while it is 1-articulated in *S.inflatus* (Steele 1967, fig. 1B); epimeral plates 2 and 3 have the posteroventral corner broadly rounded, while epimeral plates 2 and 3 are produced into a sharp tooth in *S.inflatus* (Steele 1967, fig. 1A). Additionally, the new species can be distinguished from *S.kergueleni* (Schellenberg, 1926) and *S.nipoma* (J.L. Barnard, 1961) by the presence of a toothed left lacinia mobilis, the posterior margin of gnathopod 1 is not densely setose; and the subacute posteroventral of epimeral 2 is not broadly rounded (Schellenberg 1926; Barnard 1961). Lastly, it also differs from *S.similis* by the shape of the third epimeral plate, which is neither serrated nor notched (Oldevig 1959).

## Supplementary Material

XML Treatment for Stegocephalus

XML Treatment for Stegocephalus carolus

## ﻿Additional information

### Author ORCIDs

Yanrong Wang https://orcid.org/0000-0002-7188-4232

Zhongli Sha https://orcid.org/0000-0002-2192-3758

Xianqiu Ren https://orcid.org/0009-0003-4119-397X

### Data availability

All of the data that support the findings of this study are available in the main text.
